# Miscellaneous

**Published:** 1828-06

**Authors:** 


### MISCELLANEOUS.

*Rupture of the Gut in a Hernial Sac.*—John Cox, a man between thirty and forty, was admitted into Guy's Hospital on the 16th of April, having a short time before received a kick from a horse upon a scrotal hernia, under which he had laboured for many years. His countenance was pale, the surface cold, and the pulse weak, with great prostration of strength and appearance of anxiety. He also complained of pain in the lower part of the belly, as well as in the hernial sac. Leeches were applied, and some calomel and opium administered, and he rallied a little; but the next day, the bowels refusing to act, notwithstanding the exhibition of gentle purgatives and clysters, vomiting came on, and he gradually sunk and died at six in the evening. On examination, a quantity of feculent matter was found in the abdomen, with part of the castor-oil which had been administered: these had escaped through an opening in the ileum large enough to admit the point of the little finger. Inflammation over the entire surface of the peritoneum had already taken place.

*Extensive Wound of the Abdomen.*—W. Walker, a man about forty years of age, was lately brought into the Middlesex Hospital, who had the cicatrix of a very extensive wound he had received some years previously on the abdomen. He stated that he had been a sailor on board a whale ship, and that, five years ago, having gone on deck when it was very slippery, and he having but imperfectly recovered from an injury of the foot, fell in the act of making water, so that the lower part of the abdomen came in contact with a filching knife which lay on the deck. An incision, *twelve inches and a half in length,* was the result, extending across the belly; the penis was completely divided, and the intestines protruded at each side. He bled very freely, and was confined to bed for five months; at the end of which time he was able to move about a little, and has since been able to follow his former trade, that of a stocking weaver.

---

*Strangulated Hernia, successfully treated with Tobacco Clyster.*

John Iverton, a young groom, was lately admitted at St. George's Hospital, under the care of Mr. Rose, having been subject from his childhood to a rupture on the right side, which generally went up without much trouble. It had come down about a month before his admission, and some degree of tumor remained after its apparent reduction. On the day he was admitted he had been riding, and during this time the gut came down, giving him some pain, and speedily bringing on sickness. He was bled till he fainted, put into the warm bath, had ether and ice to the tumor, and the taxis diligently applied; but without avail, the tumor remained tense and painful, extending nearly from the bottom of the scrotum, along the inguinal canal, to the inner ring; the testicle appearing to be situated below and to the inside of the swelling, and there being some effusion into the tunica vaginalis testis, which rendered the parts rather obscure. In the evening, the other remedies having been fairly tried, a tobacco clyster was administered, and speedily followed by the reduction of the hernia. Still, however, a tumor remained, which proved to be hydrocele.

---

*Fracture of the Skull, with Depression, in a Child, successfully treated.*

C. Brown, a child four and a half years old, was brought into the Middlesex Hospital, in a state of insensibility, on the 13th of March, having been run down by a horse. No contusion could be perceived on the head, but a depression was felt on examining minutely behind the right ear. On cutting down at this part, a fracture was brought into view, with depression to the depth of about a quarter of an inch. Two pieces of broken bone were so impacted as to render their removal difficult: this, however, was at length accomplished, by which a considerable portion of the dura mater was exposed. The wound was lightly dressed, and a cold lotion applied to the head. When the operation was completed, the child opened her eyes and began to cry, asking for her mother. During the ensuing night, she had a great deal of vomiting, and was restless. The bowels were kept open, and she gradually improved, so that in two days she had regained her natural appearance and manner, and very speedily recovered.

---

### *Fracture into the Knee-Joint, successfully treated.*

March 24th.—A. Aldred, a sailor, about forty years of age, fell from the top of a waggon, and broke his leg: he was brought to St. George's Hospital in two hours after, when it was found that the head of the tibia was broken off just above the insertion of the ligamentum patellæ, and the part separated was itself divided into two portions; the fibula was safe. There was a good deal of swelling, and blood appeared to be effused into the limb. This state continued to increase, and next day the leg was prodigiously swollen, with much pain, particularly about the knee.—He was twice bled, had leeches applied, and was purged; the limb was placed upon a double inclined plane, and a cold lotion constantly applied to it.

Under this treatment the symptoms gradually abated, and on the 1st of May the use of the fracture-box was discontinued, and the limb bandaged; in a week more the bandage was left off, there being only some thickening about the joint, with stiffness.

---

### *Poisoning with Oxalic Acid, successfully treated.*

A girl, only fifteen years of age, was brought into St. Thomas's Hospital on the 7th of last month, about an hour and a half after having swallowed some oxalic acid (quantity not known) for the purpose of destroying herself. She had heat and burning pain along the throat and fauces, and vomiting of bloody mucus. The first method adopted was the application of Read's syringe, by means of which the stomach was thoroughly washed out with chalk and water. After this she was so much reduced that it was necessary to give her some ammonia, with a little laudanum (ten drops,) in camphor mixture, and to apply warmth to the surface. For several days she complained of soreness of the tongue and fauces, with occasional vomiting, and tenderness on pressure at the pit of the stomach. Twelve leeches were applied to the abdomen, and a solution of the chloride of soda used as a gargle.

Under this treatment, she entirely recovered in a few days.

